# Infection dose shapes granuloma phenotypes in a humanized mouse model of tuberculosis

**DOI:** 10.21203/rs.3.rs-9849520/v1

**Published:** 2026-06-18

**Authors:** Shashi Prakash Singh, Preena Prasanna, Vinay Shivanna, Donna Perry, Edward J Dick, David F Ackart, Brendan K Podell, Riti Sharan

**Affiliations:** Texas Biomedical Research Institute; Kerala Veterinary and Animal Sciences University; Texas Biomedical Research Institute; Texas Biomedical Research Institute; Texas Biomedical Research Institute; Colorado State University; Colorado State University; Texas Biomedical Research Institute

**Keywords:** Tuberculosis, granuloma, humanized mice, immune cell recruitment, Interleukin-10, inflammation

## Abstract

Tuberculosis (TB) remains a leading cause of infectious disease mortality worldwide, yet current murine models fail to fully recapitulate human granuloma biology. Here, we utilized hCD34 + NCG humanized mice (humouse) to investigate how infection dose shapes granuloma formation and disease progression following aerosol infection with *Mtb* CDC1551. Humouse were infected with either low-dose (10^3^ CFU) or high-dose (10^6^ CFU) *Mtb* and compared with conventional C57BL/6 mice. High-dose infection induced significant weight loss, severe pulmonary pathology, and the formation of compact, highly cellular granulomas containing multinucleated giant cells, closely resembling human TB lesions. In contrast, low-dose infection produced sparse organized granulomas with reduced pathology and delayed immune recruitment. Granuloma phenotypes were independent of reconstitution efficiency, supporting the robustness of the model. Progressive disease was associated with depletion of hCD8 + T cells and hNKT cells, mirroring immune signatures observed in human TB. Low-dose infection promoted distinct immunoregulatory responses characterized by pulmonary MIP-3α, IL-17A, and IL-15 signaling together with systemic IL-10 induction. IHC and RNAscope analyses revealed dose-dependent macrophage accumulation and actively replicating bacilli within granulomatous lesions. Collectively, these findings demonstrate that infection dose is a major determinant of granuloma heterogeneity in the humouse model.

## Introduction

Despite the availability of effective anti-tubercular drug therapy, tuberculosis (TB) remains one of the leading causes of infectious disease-related morbidity and mortality worldwide. According to the World Health Organization (WHO), an estimated 10.7 million individuals developed TB and approximately 1.23 million deaths occurred in 2024 alone. Progress toward TB control has been further hindered by the COVID-19 pandemic which disrupted TB surveillance systems, delayed diagnosis and treatment, and impeded clinical trials^1^. *Mycobacterium tuberculosis* (*Mtb*), the etiological agent of TB, establishes infection primarily in the lungs, where it forms granulomas, the pathological hallmark of the disease. Granuloma formation represents a complex, multistage host immune response aimed at containing the pathogen. However, this structure also paradoxically provides a protected niche in which *Mtb* can persist in a latent state, or reactivate, leading to disease progression and dissemination^2^. Thus, granulomas function as dynamic microenvironments that balance host protection with bacterial survival^3^.

Structurally, granulomas are highly organized lesions composed of giant foamy macrophages, including multinucleated giant cells, often accompanied by neutrophils with *Mtb* bacilli at the core. These infected macrophages are encircled by concentric layers of immune cells, predominantly T and B lymphocytes^4^, forming a characteristic lymphocytic cuff. Effective granuloma formation is critical for restricting bacterial spread, whereas failure is associated with uncontrolled infection. The granuloma diversity encompasses variations in cellular composition, spatial architecture, and functional outcomes^5, 6^. Understanding the determinants of granuloma heterogeneity is therefore central to advancing TB research. While host immune responses and bacterial factors have been implicated, the role of infection dose in shaping granuloma phenotype remains poorly defined. Furthermore, although murine models have provided valuable insights into TB pathogenesis, they do not fully recapitulate the complexity of human disease. In this study, we address these gaps by investigating how varying infection doses influence granuloma formation and phenotypic diversity in a humanized mouse model of TB, with the goal of elucidating key drivers of granuloma heterogeneity and their implications for disease outcome.

*Mtb* pathogenesis has been extensively investigated using a range of animal models, including mice, rabbits, guinea pigs, and non-human primates (NHPs)^7^. Among these, murine models are the most widely used due to their affordability, genetic tractability and extensive availability of immunological tools. However, conventional mouse strains fail to fully recapitulate key features of human TB, particularly the formation of structurally and functionally representative granulomas. While the C3HeB/FeJ (Kramnik) mouse develops necrotic lesions that partially resemble human pathology, the model shows macrophage dysfunction, impaired nitric oxide production and limited B cell responses, thereby restricting its ability to faithfully model the complexity of human granulomas^8, 9^. In contrast, NHP models more closely mirror human disease^3, 10^, including granuloma heterogeneity and immune responses^11^, but their use is constrained by higher costs, ethical considerations, and limited genetic and immunological resources. Collectively, these limitations underscore a critical need for alternative models that better capture human-specific immune responses and granuloma biology.

Humanized mouse (humouse) models have emerged as a promising platform to bridge this gap^12^. These models are engrafted with human hematopoietic cells and develop functional human immune cells, including human T cells (CD3^+^), T-helper cells (CD3^+^CD4^+^), cytotoxic T cells (CD3^+^CD8^+^), B cells (CD19^+^), macrophages (CD14^+^), and natural killer cells (NKG2A^+^), enabling the production of human-specific cytokines and signaling networks^13–15^. Recent work has demonstrated that humouse models can support *Mtb* infection and generate granuloma-like structures with human cell infiltration, providing important insights into human immune responses^16, 17^. However, the extent to which key variables, such as infection dose, shape granuloma architecture, immune recruitment, and disease trajectory in these models remains largely unexplored.

In this study, we address this gap by investigating how infection dose influences granuloma phenotype and immune responses in a humouse model of TB. We utilized a novel NOD-*Prkdc*^*em26Cd52*^*Il2rg*^*em26Cd22*^/NjuCrl coisogenic, genetically engineered triple-immunodeficient model intravenously engrafted with hCD34^+^ hematopoietic stem cells (HSCs) to study the recruitment and distribution of human immune cells in lungs^18–20^. We hypothesized that variation in infection dose would differentially modulate immune recruitment and granuloma organization. Consistent with this hypothesis, humouse exhibited dose-dependent differences in disease progression and immune responses. Unlike conventional C57BL/6 mice, which are relatively resistant to *Mtb* infection, humouse displayed human-like susceptibility, including progressive weight loss and disease pathology. High-dose infection induced rapid morbidity, robust immune cell recruitment, and severe tissue pathology, whereas low-dose infection resulted in delayed cellular recruitment, reduced pathology, and prolonged survival. Importantly, we identified MIP-3a, IL-17A and IL-15 in modulating immune cell recruitment responses, particularly under low-dose conditions, where they heighten inflammation in pulmonary tissue while IL-10 suppresses the immune system at the systemic level, consistent with human clinical observations^21^. Together, our findings extend prior work on humouse TB models by demonstrating that infection dose is a critical determinant of granuloma phenotypes and immune dynamics.

## Results

### 1. Infection dose drives early clinical manifestations in humouse TB model.

**Figure 1:**
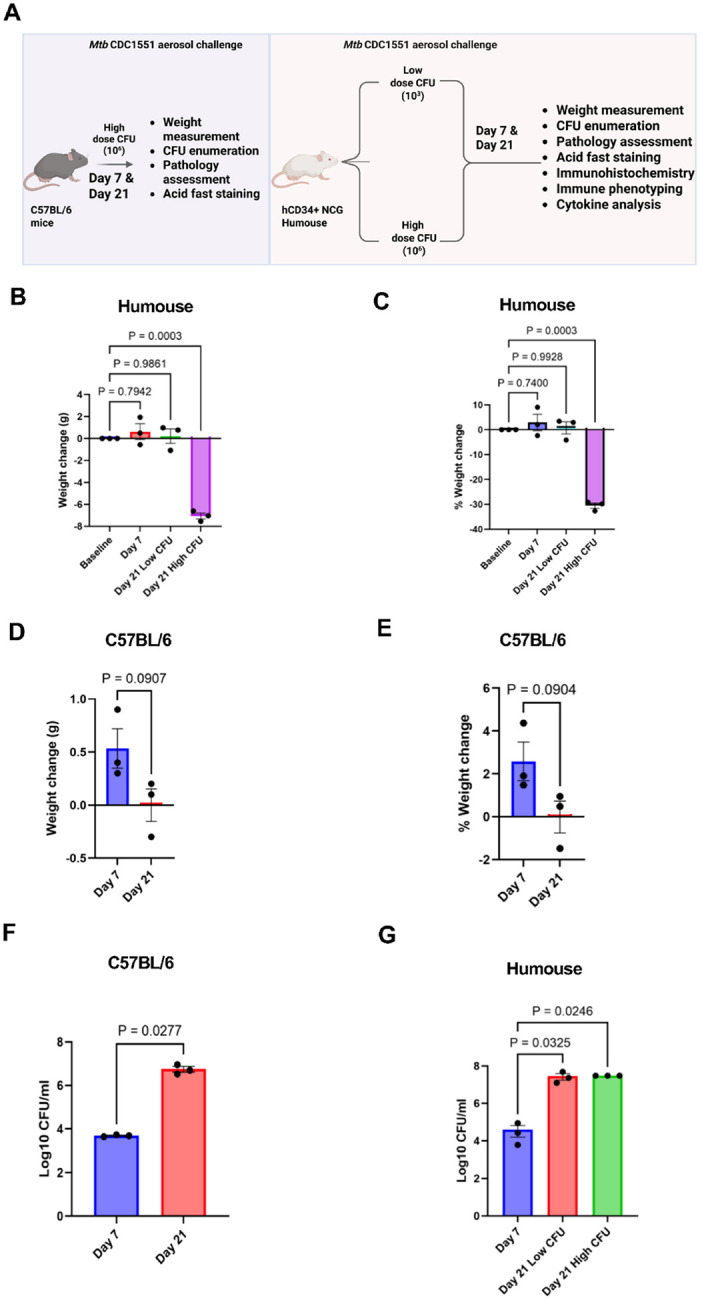
**(A)** Study design representing experimental protocol. 3–5 mice per group, C57BL/6 mice and NCG-hCD34+ humouse were infected until 7 days and 21 days post-infection. Weights were measured weekly and at each timepoint bacterial survival, pathology and immune phenotyping in the lung were performed. The body weight was measured and plotted as a change in weight (**B**) and a percentage change (**C**) in weight after *Mtb*aerosol challenge. Results are shown as mean ± SEM. The data acquired were used in One-way ANOVA with Tukey’s post-hoc test. No significant weight change occurred in non-infected baseline mice with the low-dose CFU group mice. The weight change was significantly decreased in the high-CFU group humouse when compared with the baseline uninfected mice. (**D & E**) shows the C57BL/6 mice's weight and percentage weight after high-dose infection. The data acquired were used in an unpaired t-test. (**E &F**) Bacterial survival at day 7 and day 21 of C57BL/6 mice and day 7 and day 21 of low and high CFU infection dose in hCD34+ NCG humouse. Right upper and middle lobes of humouse were homogenized and plated on a 7H11 plate with OADC with antibiotics. After 4 weeks, colonies were counted. The data acquired were used in an unpaired t-test for C57BL/6 mice and One-way ANOVA with Tukey’s post-hoc test for humouse *Mtb* infection. Significance levels are indicated as *P < .05, **P < .01, ***P < .001, and ns as non-significant.

Granuloma formation is a defining feature of TB pathogenesis; however, granulomas formed in conventional C57BL/6 mice differ substantially from those observed in humans^9, 22, 23^. To better model human disease, we employed the hCD34^+^ NCG humouse model and compared its response to infection with that of the widely used C57BL/6 mouse model. C57BL/6 mice were challenged with a high dose (10^6^ CFU), while humouse were infected with either low (10^3^ CFU) or high (10^6^ CFU) doses of *Mtb* CDC1551 via aerosol. Granuloma formation and disease progression were evaluated at days 7 and 21 post-infection. At each time point, 3–5 mice per group were euthanized, and lung tissues were collected for histopathological analysis, bacterial burden quantification, acid-fast staining, immunohistochemistry, immunophenotyping, and cytokine profiling (Fig. 1A). The infection dose had a pronounced effect on clinical outcomes. Consistent with prior reports^13^, humouse in the low-dose group exhibited minimal, non-significant changes in body weight. In contrast, high-dose infection resulted in significant weight loss (*p* =0.003), with animals losing approximately 30% of their initial body weight (Fig. 1B, C). Notably, in C57BL/6 mice, body weight typically remains stable until at least day 21 following aerosol infection (Fig.1D, E). In addition to weight loss, we observed that humouse displayed reduced movement compared to C57BL/6 controls, further indicating increased disease severity. We further evaluated the bacterial burden in C57BL/6 as well as humouse, both groups showed increased *Mtb* burden at day 21 (p<0.05) (Fig. 1F, G). There was no difference in survival of C57BL/6 mice and humouse (**Fig. S1A, B**). Together, these findings demonstrate that the humouse model exhibits heightened susceptibility to *Mtb* infection relative to conventional murine models, and that infection dose is a key determinant of early clinical manifestations.

### 2. Infection dose drives distinct granuloma phenotypes and pulmonary pathology.

**Figure 2:**
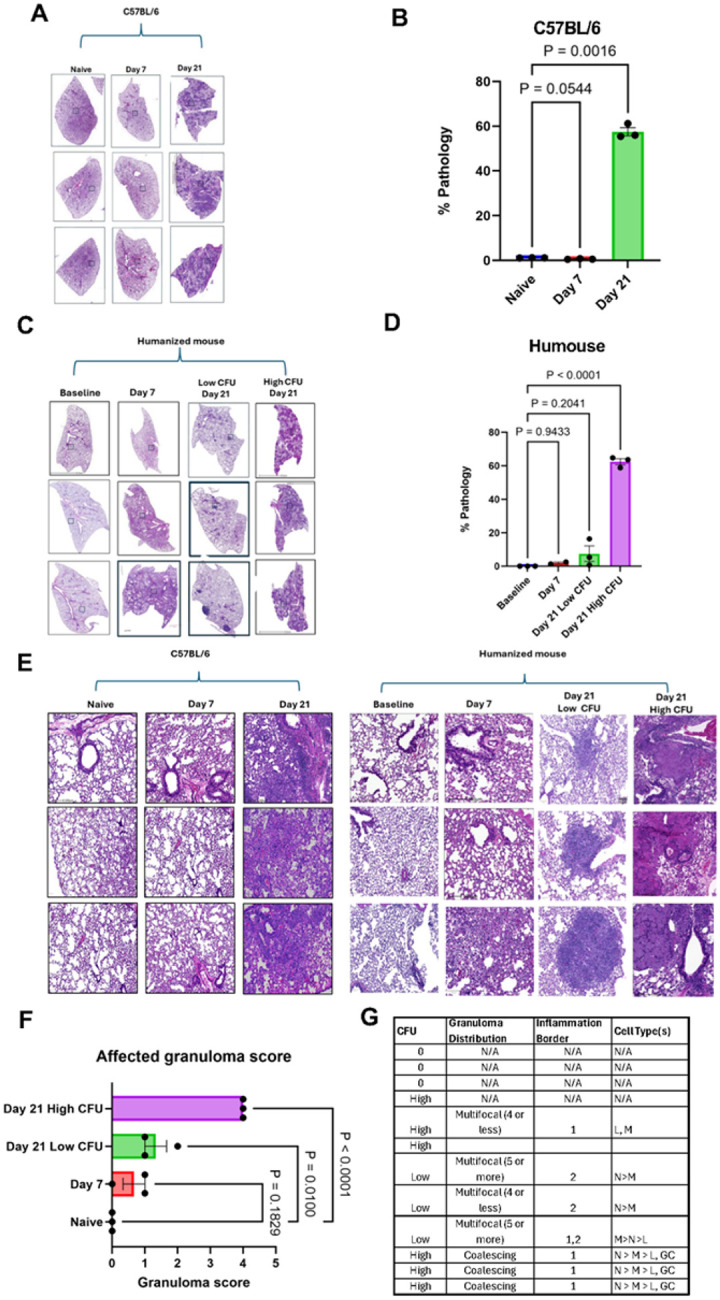
Histopathological analysis of lungs in C57BL/6 mice and humouse. H&E staining (**A**) was performed to detect histopathology changes that occurred due to *Mtb*infection at Day 7 and Day 21 and compared with the naïve mice. A representative figure of each mouse at the indicated time point is shown, scalebar, 1mm. HALO 4.0 was used to quantify lesions or cellular aggregates in the lungs of day 7 and Day 21 *Mtb* infected mice and compared with the naïve mice (**B**). Results are shown as mean ± SEM. The data acquired were used in One-way ANOVA with Tukey’s post-hoc test. Lung sections of naïve day 7, low and high-CFU groups at day 21 showed remarkably distinct necrotic debris when compared to naïve humouse (**C**). A representative figure of each humouse at the indicated time point is shown, scalebar, 1mm. HALO 4.0 was used to quantify lesions or cellular aggregates to assess the percentage of inflammation (**D**). Data acquired after analysis are shown as mean ± SEM. The data acquired were used in One-way ANOVA with Tukey’s post-hoc test. Magnified image (100 μm) of lungs shows distinct atypical granuloma phenotypes in low and high-CFU groups (**E**). In the high-CFU group, many diffuse cellular aggregates are present while the low-CFU group shows fewer cellular aggregates but clear boundaries. (**F**) Shows the granuloma-affected score in different groups. Granuloma score was calculated using focal (<10%), multifocal (<25%) for less than 4, multifocal (<50%) for 5 or more, and coalescing (<75%). Results are shown as mean ± SEM. The data acquired were used in One-way ANOVA with Tukey’s post-hoc test. Detailed histopathology (**G**) description of lungs of different groups showing distribution, inflammatory border and cell types. Significance levels are indicated as *P < .05, **P < .01, ***P < .001, and ns as non-significant.

To determine how infection dose influences granuloma formation, we compared pulmonary pathology in C57BL/6 mice and humouse. H&E staining revealed minimal cellular infiltration in naïve C57BL/6 mice and at day 7 post-infection across all groups. By day 21, C57BL/6 mice developed typical diffuse murine granulomas, whereas humouse displayed striking dose-dependent differences in lesion architecture (Fig. 2A, B). Histopathological analysis showed that low-dose infection induced sparsely organized granulomas with limited cellular recruitment. High-dose infection in humouse resulted in extensive pulmonary pathology characterized by numerous compact and well-demarcated granulomas (Fig. 2C, D). At 100x magnification, C57BL/6 mice showed diffused cellular aggregates while discrete granulomatous lesions were apparent in the high-CFU group but remained less prominent in low-dose infected humouse (Fig. 2E). Quantitative histopathological assessment further demonstrated that by day 21, low-dose infected humouse developed four or fewer multifocal granulomas per lung section, whereas high-dose infected humouse exhibited more than five multifocal granulomas (Fig. 2F). Importantly, granulomas in the high-CFU group showed marked neutrophil and monocyte infiltration together with multinucleated giant cells which is a hallmark feature of human TB pathology, which was less evident in the low-CFU group (Fig. 2G; Table 1). Together, these findings demonstrate that infection dose is a critical determinant of granuloma organization, inflammatory cell recruitment, and pathological severity in the humouse TB model.

### 3. Human immune reconstitution does not alter granuloma-associated responses.

**Figure 3:**
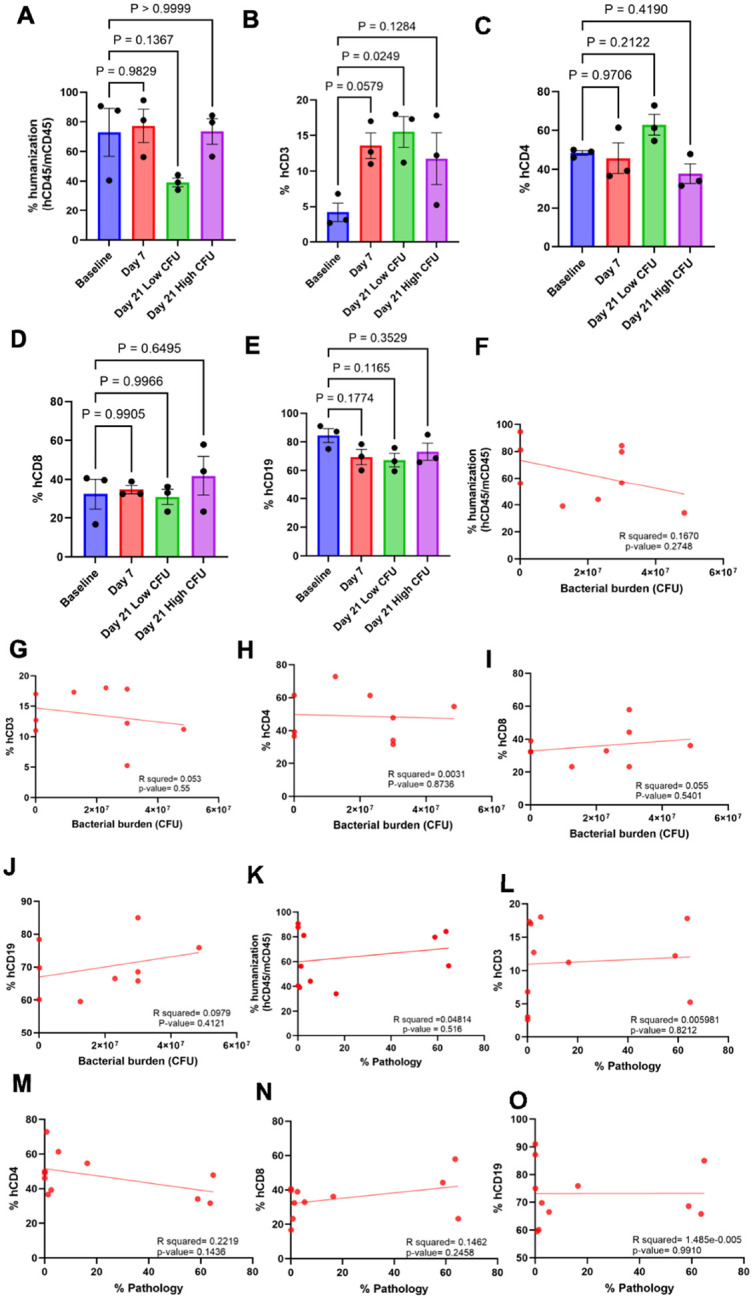
Humanization ratio (hCD45/mCD45) is critical for inducing the human-specific immune response in humouse. Multiplex flow cytometry was used to identify human CD45+ leukocytes. Further hCD3+, hCD3+hCD4+, hCD3+ hCD8+, hCD19 cells were quantified. The gating strategy we used to identify was FSC/SSC leukocytes → hCD45^+^/mCD45^+^ leukocytes → hCD3^+^ T cells/hCD19^+^ B cells → hCD4^+^ T cells and hCD8^+^ T cells. The data represent means ± SEM. The data acquired were used in one-way ANOVA with Tukey’s post-hoc test. Linear regression analysis of bacterial survival (CFU) in lungs vs % humanization (**F**), %CD3 (**G**), %CD4 (**H**), %CD8 (**I**) and %CD19 (**J**). R-squared was calculated as the goodness of fit. Statistical significance was calculated using the p-value for the slope coefficient in a linear regression model using Graphpad Prism (11.01 version). Fig. 3L to O shows linear regression analysis between % pathology vs % humanization, %CD3, %CD4, %CD8 and %CD19 respectively. R-squared was calculated as the goodness of fit. Significance levels are indicated as *P < .05, **P < .01, ***P < .001, and ns as non-significant. point.

Human TB granulomas differ markedly from those formed in conventional C57BL/6 mice models^23^. Because humouse are generated from individual donors and exhibits variable levels of immune cell engraftment, we investigated whether humanization efficiency influenced granuloma formation. Flow cytometric analysis demonstrated no significant differences in the humanization ratio (hCD45/mCD45) among experimental groups (Fig. 3A). Similarly, frequencies of hCD3^+^ (Fig. 3B), hCD4^+^T (Fig. 3C), hCD8^+^T (Fig. 3D) and hCD19^+^ (Fig. 3E)were not associated with differences in overall pathology. We measured the correlation between *Mtb* bacterial burden vs percentage humanizations (hCD45+/mCD45+) (Fig. 3F), %hCD3 (Fig. 3G), % hCD4+(Fig. 3H), %hCD8(Fig. 3I) and %hCD19(Fig. 3J) respectively. Similarly, we measured the impact of % pathology to the % humanization (Fig. 3K), % hCD3 (Fig. 3L), % hCD4+(Fig. 3M), % hCD8 (Fig. 3N), and % hCD19(Fig. 3O) respectively. The correlation analysis revealed no significant association between % human leukocytes engraftment levels and bacterial burden, % pathology in the lung. Together, these findings indicate that variability in human reconstitution does not substantially influence the pulmonary immune response in this model, supporting the robustness and reproducibility of the humouse system for studying TB pathogenesis.

### 4. *Mtb* infection drives depletion of CD8^+^ T and NKT cells, recapitulating human TB immune signatures.

**Figure 4:**
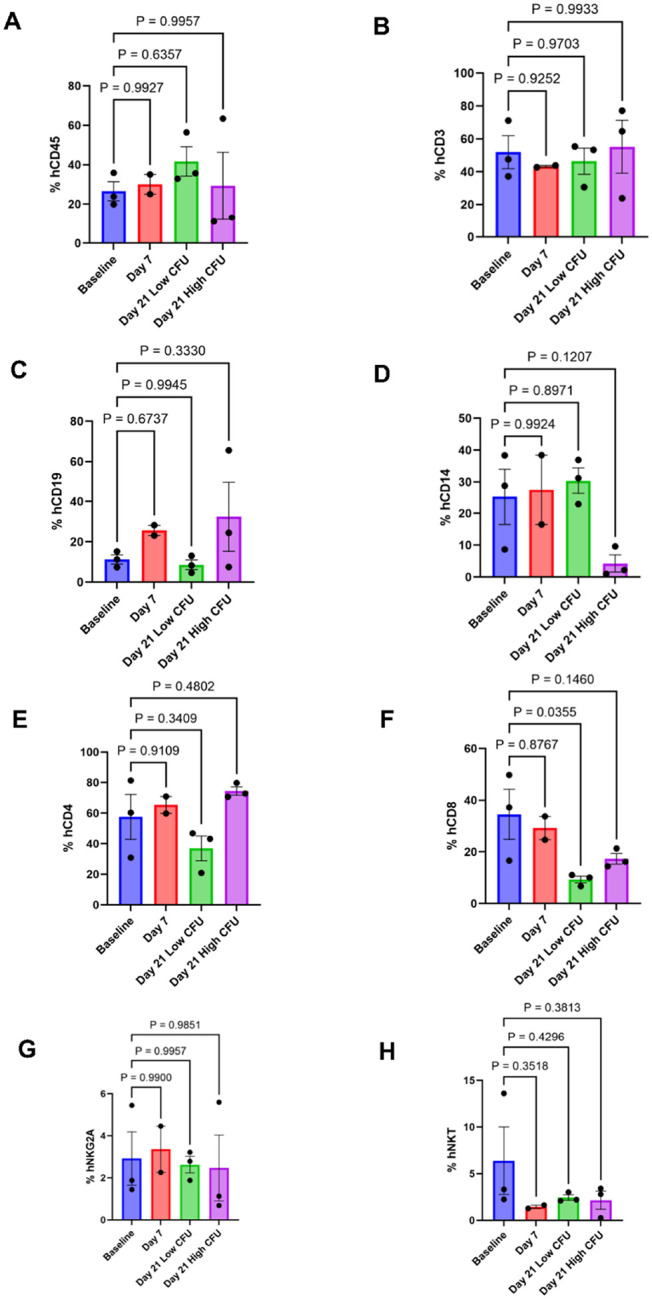
Immune cell phenotypes were quantified with or without *Mtb* infection. The gating strategy utilized to identify cells was FSC/SSC leukocytes → hCD45^+^/mCD45^+^ leukocytes → hCD3^+^ T cells/hCD19^+^ B cells → hCD4^+^ T cells and hCD8^+^ T cells, NKG2A+, NKT cells. Data represent means ± SEM. The data acquired were used in one-way ANOVA with Tukey’s post-hoc test. Significance levels are indicated as *P < .05, **P < .01, ***P < .001, and ns as non-significant.

To further define the human immune landscape associated with dose-dependent granuloma formation, we characterized pulmonary human immune cell populations during *Mtb* infection using multiparameter flow cytometry (**Fig. S2**). Consistent with our previous findings, the overall humanization ratio (hCD45^+^/mCD45^+^) remained stable across experimental groups (Fig. 4A), indicating that differences in disease severity were not driven by variability in human immune reconstitution. Similarly, no significant differences were observed in total hCD3^+^ T cells (Fig. 4B), hCD19^+^ B cells (Fig. 4C), or hCD14^+^ myeloid populations (Fig. 4D). Despite comparable overall human immune engraftment, distinct lymphocyte dynamics emerged during infection. Humouse exhibited greater pulmonary infiltration of hCD4^+^ T cells relative to hCD8^+^ T cells (Fig. 4E, F), associated with active granulomatous inflammation. Notably, low-dose infection was associated with a significant ~20% depletion of hCD8^+^ T cells (*p*= 0.035), coinciding with reduced pulmonary pathology (Fig. 4F). Given the emerging role of innate lymphocytes in TB immunity, we additionally evaluated NK and NKT cell populations. Although hNK cells showed a modest, non-significant increase following infection (Fig. 4G), hNKT cells were depleted (~10%, *p* = 0.38) irrespective of infection dose when compared to naïve controls (Fig. 4H). Importantly, this reduction mirrors observations reported in human TB patients, further supporting the ability of the humouse model to recapitulate clinically relevant immune features of TB. Together, these findings demonstrate that dose-dependent TB pathology in humouse is accompanied by selective depletion of hCD8^+^ and hNKT cell populations, revealing human-like immune dysregulation associated with progressive granulomatous disease.

### 5. Low burden *Mtb* infection induces systemic immune regulation and innate chemokine signalling.

**Figure 5:**
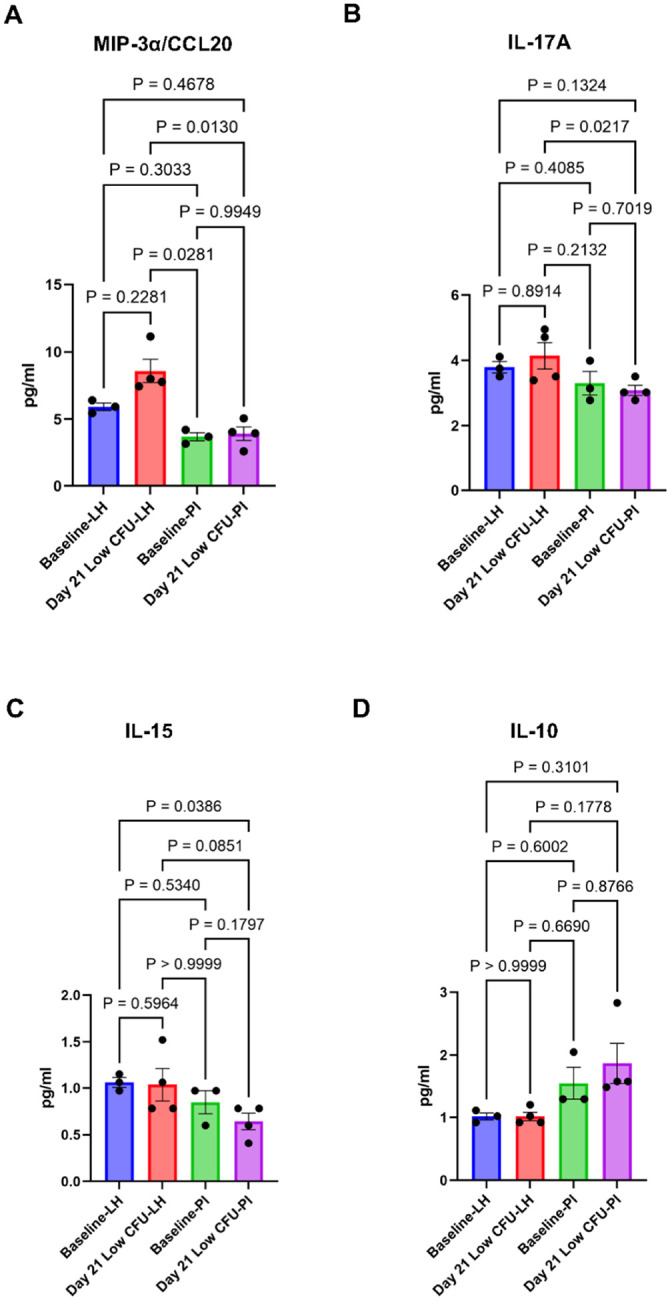
Cytokines were analyzed in lung homogenates (LH) and plasma (Pl) of humouse. We used the Millipore 25 plex Th17 cytokine panel to screen and quantify the various cytokines (**Fig. S3**). MIP-3α (**A**), IL-17A (**B**), and IL-15 (**C**) were found elevated in lung homogenate compared to plasma while hIL-10 (**D**) was found high in plasma, it did not change in lung homogenate supernatant. Data represent means ± SEM. The data acquired were used in one-way ANOVA with Tukey’s post-hoc test. Significance levels are indicated as *P < .05, **P < .01, ***P < .001, and ns as non-significant.

To define the immunoregulatory landscape associated with low-dose aerosol infection, we performed multiplex cytokine profiling in plasma and lung tissue from infected humouse (**Fig. S3 A-I, S4A-K**). Among the analytes examined, we observed increased expression of Macrophage Inflammatory Protein-3 alpha (MIP-3α/CCL20), a chemokine implicated in the recruitment of innate immune populations, including macrophages, dendritic cells, and CCR6^+^ leukocytes, to sites of infection and tissue inflammation. MIP-3α levels in lung homogenate (LH) trended significantly higher (*p*=0.013) than plasma concentrations (Fig. 5A), potentially reflecting localized chemotactic gradients within infected lung tissue. IL-17A, a critical proinflammatory cytokine, induces aberrant inflammation in the lung after *Mtb* infection. Though not significant ((*p*= 0.021), IL-17A (Fig. 5B) and IL-15 levels (Fig. 5C) were marginally higher in lung homogenate than plasma. IL-15 enhances protective immune responses against *Mtb* infection, highlighting its importance in pulmonary compartment. Interleukin-10 (hIL-10) was marginally elevated in plasma compared to lung homogenate following low-dose infection, though this difference was not significant (*p*=0.1778) (Fig 5D). The discordance between circulating and pulmonary IL-10 suggests spatial compartmentalization of immune regulation during early granuloma development. In parallel, collectively, these findings suggest that low-dose aerosol infection establishes a distinct immunoregulatory environment characterized by pulmonary chemotactic, pro-inflammatory signalling, and systemic IL-10 induction. In contrast to the severe granulomatous pathology observed following high-dose challenge, low-dose infection may promote a more controlled inflammatory program that supports early innate immune recruitment while limiting excessive tissue-destructive responses. These data support the concept that granuloma phenotype in the humouse model is shaped by dosedependent immune polarization.

### 6. Immunohistochemical analysis reveals dose-dependent macrophage accumulation within granulomatous lesions.

**Figure 6:**
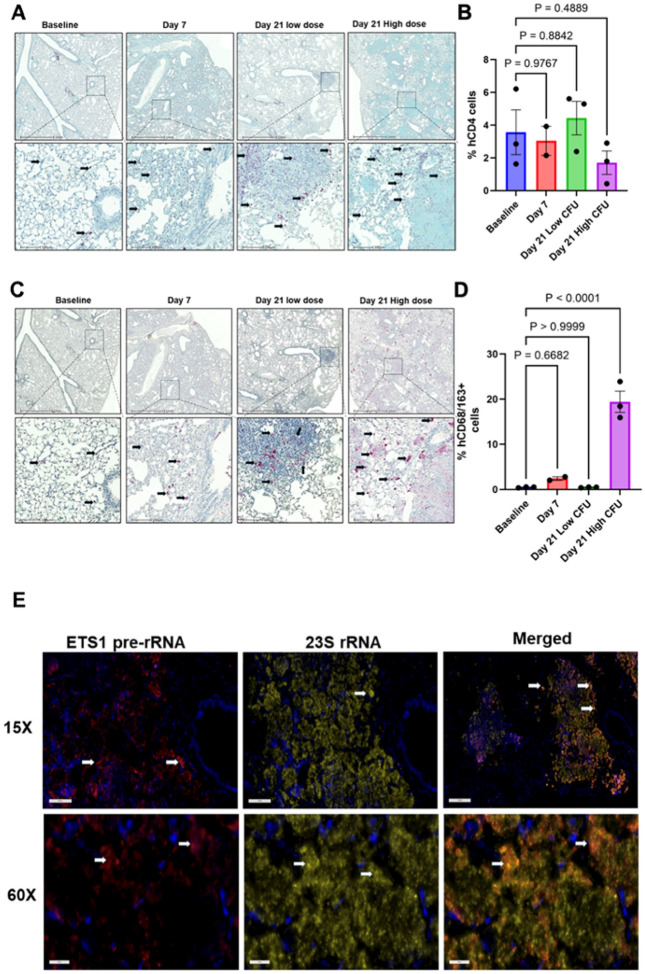
hCD4^+^ T-cell and hCD68/163^+^ macrophage populations in the lung. Immunohistochemistry staining was performed on lung tissue sections. Fast Red chromogen was used to stain hCD4^+^ cells **(A)**, with corresponding HALO quantification **(B)**. Similarly, hCD68/163 expression is shown **(C)** at 1 mm, with corresponding HALO quantification **(D)** demonstrating significant macrophage recruitment in the lungs of high-dose humouse-infected animals at day 21. Lower panels show magnified images (100 μm) of the corresponding upper panels. RNAscope was performed on lung sections to detect replicating and non-replicating *Mtb* **(E).**Pre-rRNA labelled with Opal 690 was used to identify replicating *Mtb*, while 23S rRNA labelled with Opal 570 was used as a marker for non-replicating *Mtb*. Black and white arrows indicate stained cells. Data are presented as mean ± SEM. Statistical analysis was performed using one-way ANOVA with Tukey’s post hoc test. Significance levels are indicated as *P* < 0.05, *P < 0.01, **P < 0.001, and ns (not significant).

To characterize the spatial organization and cellular composition of pulmonary granulomas during *Mtb* infection, we performed chromogenic immunohistochemistry (IHC) on lung tissue sections to assess the recruitment and localization of human hCD4^+^ T cells and hCD68/163^+^ macrophages. Analysis of hCD4^+^ T-cell staining revealed no significant differences in overall cellular abundance across experimental groups (Fig. 6A, B), although a modest trend toward increased hCD4^+^ T-cell recruitment was observed following low-dose infection. Spatially, hCD4^+^ T cells were predominantly localized to perivascular regions (black arrows), consistent with organized lymphocytic clustering adjacent to inflammatory lesions. In contrast, hCD68/163^+^ macrophage staining demonstrated marked and significantly elevated macrophage accumulation in the day 21 high-dose infection group relative to all other conditions (Fig. 6C, D). These macrophages were densely associated with granulomatous inflammatory regions (black arrows), supporting the development of highly cellular and organized lesions during progressive disease. To further assess bacterial viability and replication within granulomatous tissue, we performed RNAscope analysis on lungs from the day 21 high-dose group. Extensive colocalization of pre-rRNA and 23S rRNA signals (yellow) was observed within pulmonary lesions, indicating the presence of metabolically active and replicating *Mtb* populations at day 21 post-infection (Fig. 6E, S5). Together, these findings demonstrate that progressive high-dose infection is associated with enhanced macrophage recruitment and persistence of actively replicating bacilli within granulomatous lesions, highlighting the development of a highly inflammatory granuloma microenvironment in the humouse model.

## Discussion

Understanding the immunologic and pathological determinants that govern granuloma formation remains a major challenge in TB research, particularly given the limitations of conventional murine models in recapitulating human TB pathology. In this study, we demonstrate that the hCD34 + NCG humouse model develops dose-dependent pulmonary granuloma phenotypes that closely resemble key features of human TB. A central finding of this study is that infection dose profoundly influences granuloma architecture and pathology in this model. Low-dose aerosol infection induced sparse and poorly organized lesions whereas high-dose challenge promoted the formation of compact, highly cellular granulomas accompanied by marked pulmonary pathology and clinical deterioration. We noted that high-dose infected humouse contained dense monocyte/macrophage infiltrates that are rarely observed in conventional C57BL/6 models but are characteristic of human TB lesions. These findings support the concept that granuloma organization is not only a consequence of bacterial presence but also reflects the magnitude and quality of host immune response elicited during infection.

We also noted that differences in granuloma phenotype were not attributable to variability in human immune engraftment. Despite the inherent donor-to-donor variability, overall humanization levels and major lymphocyte populations remained comparable across experimental groups. This observation supports the notion that the divergent pathological outcomes are driven primarily by infection burden and associated immune polarization and not differences in baseline immune reconstitution. Our data also revealed that progressive granulomatous disease is associated with selective depletion of pulmonary hCD8 + T cells and hNKT populations. Reduced CD8 + T-cell abundance coincided with enhanced granuloma organization and severe pulmonary inflammation in high-dose infected animals. This is consistent with reports linking impaired cytotoxic lymphocyte responses in progressive human TB. Depletion of hNKT cells mirrors observations in TB patients in that innate-like myeloid populations contribute critically to antimycobacterial immunity. This suggests that severe granulomatous pathology in humouse model is accompanied by disruption of immune populations involved in cytotoxic control and immune homeostasis.

Low-dose infection induced a distinct immunoregulatory profile characterized by systemic hIL-10 production and pulmonary CCL20, IL-17A and IL-15 signaling. Elevated circulating IL-10 in the absence of significant pulmonary induction suggests compartmentalized immune regulation during early infection. It potentially reflects mechanisms that limit excessive inflammatory pathology while preserving tissue integrity. Concurrent increases in CCL20, IL-17A and IL-15 further support active recruitment of innate immune populations involved in early granuloma establishment. These findings suggest that the low-dose infection promotes a more controlled inflammatory microenvironment that differs fundamentally from the highly inflammatory granuloma program observed following high-dose challenge. Immunohistochemical and RNAscope analyses provided spatial context to these immune dynamics. High-dose infection was associated with dense accumulation of hCD68/163 + macrophages within granulomatous lesions. This indicates that these inflammatory macrophage- rich granulomas remain permissive to ongoing bacterial replication. This observation aligns with the concept that granulomas can serve as both protective and permissive niches for *Mtb* persistence. Detection of the *Mtb* organism by RNAScope labelling of the mature 23S rRNA revealed a dense frequency of bacilli localizing to the necrotizing lesions and neutrophil-associated debris characteristic of the high-dose challenge. This location indicates that the bacilli are largely present in the extracellular space among non-viable inflammatory cells. In other murine models with necrotizing inflammation, such as the C3HeB/FeJ mouse^24^, this extracellular microenvironment is also the location of the majority of bacilli. However, the high-dose humouse model is distinctly different in that these extracellular bacilli exist in a state of high replication, in contrast to the quiescent non-replicative state in primary necrotic granulomas of the C3HeB/FeJ mouse^25^. This represents an atypical manifestation since organisms present among host necrosis typically exist in a state of dormancy.

Several limitations of this study should be acknowledged. The relatively short infection period captures early granuloma development rather than chronic disease progression. Larger longitudinal studies will be necessary to define how these immune programs evolve over time. In addition, although the humouse model recapitulates multiple hallmarks of human TB, incomplete maturation of certain human immune subsets remains an inherent limitation of the current humouse systems. Future studies incorporating single-cell and spatial transcriptomic approaches may further define the cellular networks that govern granuloma evolution in this model. In summary, our findings demonstrate hCD34 + NCG humouse model reproduces key pathological features of human TB in a dose-dependent manner. These results highlight the humouse model as a valuable translational platform for dissecting human TB granuloma biology.

## Methods

### Ethics statement

The study was approved by the Institutional Animal Care and Use Committee (IACUC). All experiments and procedures were performed in accordance with IACUC 1887MU. Animals were housed in a centralized research animal facility accredited by the Association for Assessment and Accreditation of Laboratory Animal Care (AAALAC) International. All experimental protocols were perfomed in BSL3 facility.

### NCG Humouse

All CD34^+^ NCG Humouse were procured from Charles River Laboratories. At 12 weeks of age, mice were irradiated with gamma radiation and subsequently infused with human CD34^+^ hematopoietic stem cells from 3–5 donors. Mice were considered ready for experimentation once the humanization ratio (engraftment) exceeded 25%, as tested by flow cytometry of immune cell subsets in PBMCs. All humouse were between 18–19 weeks old at the beginning of the study, appeared healthy, and were utilized in three independent experimental repeats. We included 3–5 mice per time point for each experiment. All animal procedures were conducted in Animal Biosafety Level 3 (ABSL3) facilities at the Texas Biomedical Research Institute, San Antonio, Texas.

### Mtb infection

A Glas-Col aerosol chamber was used to expose humouse to two different doses of *Mtb* CDC1551. Targeted doses were set at 10^3^ CFU for low and 10^6^ CFU for high colony forming unit (CFU) per group. Briefly, the target CFU was prepared by diluting 1 ml of bacterial culture into 4 ml of Ultrapure water. This 5 ml solution was loaded into the nebulizer and ran at preset conditions for 30 minutes. Following exposure, the chamber surfaces and cages were sterilized with Ultraviolet (UV) radiation for 20 minutes. After the challenge, mice were transferred to sterile cages with *ad libitum* access to food and water.

### CFU enumeration

Following *Mtb* exposure, mice were euthanized on day 0, day 7 and day 21. Upper 2 right lung lobes were aseptically excised and homogenized at 20 sec cycles for one time at a standard manufacturer’s (Precelly’s) protocol in 1 ml of complete RPMI-1640 (11875–093, Gibco). After homogenization, serial dilutions in Ultrapure water (10977–015, Invitrogen) were used to prepare 10^1^, 10^2^, 10^3^, 10^4^ and 10^5^ by starting from the neat lung homogenate. The dilutions were plated on 7H11 Middlebrook agar (cat. no. M0428, Millipore), containing oleic acid (cat. no. S25451A, Fisher), dextrose (cat. no. BP350, Fisher), catalase (cat. no. C1328, Spectrum) and antibiotics containing Trimethoprim (cat. no. T0667, Sigma), Carbenicillin (cat. no. C1389, Sigma), Polymyxin B (cat. no. P1004, Siga), Cycloheximide (cat. no. C7698, Sigma).

### Lung tissue isolation and processing for flow cytometry

The left lung lobe was aseptically removed and homogenized using a Miltenyl Biotec dissociator to be processed for flow cytometry. The homogenized tissue was incubated with Collagenase II (cat. no. C2674, Sigma) and Dispase at 37°C for 30 minutes to digest the pulmonary extracellular matrix. Following digestion, cells were washed with RPMI-1640 media containing Fetal Bovine Serum (FBS) (cat. no. A5670701, Gibco) and Penicillin-Streptomycin (cat. no. 15140–122, Gibco). The cells were treated with RBC lysis buffer (cat. no. A10492–01, Gibco). The reaction was inactivated by FBS. The resulting cell suspension was filtered through a 100-micron nylon mesh (cat. no. 431752, Milipore), resuspended in 1 ml of RPMI-1640 complete media. Live and dead cells were counted using a Bio-Rad Countess automated cell counter.

### Flow cytometry

Flow cytometry was performed on lung cells at day 7, and day 21 post-infection time points. Briefly, 1 million lung cells were washed with 1X PBS (cat. no. 21–040cv, Corning), aliquoted into tubes, and incubated with a Live/Dead Fixable Dead Cell Stain (APC-eFluor 780, Thermo Fisher). The cells were then stained with the following antibody panel: human CD45 (clone Hi30, Pacific Blue, BioLegend), mouse CD45 (clone 30F11, BV711, BioLegend), human CD3 (clone UCHT1, BV650, BioLegend), human CD4 (clone OKT4, Alexa Fluor 647, BioLegend), human CD8 (clone SK1, PE-Dazzle 594, BioLegend), human CD19 (clone HIB19, FITC, BioLegend), NKG2A (clone S19004C, PE, BioLegend) and human CD14 (clone 26ic, PerCP-Cy5.5, Caprico). After a 30-minute incubation, cells were washed with MACS buffer (cat. no. 130-091-24, Miltenyl Biotec), resuspended in 250 μl of BD Fixation and Stabilization buffer (cat. no. 338036, BD), and acquired on a BD FACSymphony^™^ A3. Data were analyzed using FlowJo software (version 10.10.0, BD Life Sciences) according to a defined gating strategy. The gating strategy utilized to identify cells was FSC/SSC leukocytes → hCD45^+^/mCD45^+^ leukocytes → hCD3^+^ T cells/hCD19^+^ B cells → hCD4^+^ T cells and hCD8^+^ T cells, NKG2A+, NKT cells (**Fig. S2**).

### Cytokine analysis

A 25-plex premixed cytokine panel was purchased from Millipore (Milliplex MAP Human Th17 Magnetic Bead Panel, cat. No. HT17MG-14K-PX25, Millipore). The assay was performed according to the manufacturer’s instructions. Plasma samples and lung homogenates collected from humouse were analyzed. All samples were centrifuged in an Accu Spin Micro 17 (Fisher Scientific) at 300 x g for 5 min to remove any debris prior to dilution (1:2) in PBS. The analytes included in the panel were: IL-17F, GM-CSF, IFN-γ, IL-10, CCL20 (MIP-3α), IL-12p70, IL-13, IL-15, IL-17A, IL-22, IL-9, IL-1β, IL-33, IL-2, IL-21, IL-4, IL-23, IL-5, IL-6, IL-17E (IL-25), IL-27, IL-31, TNF-α, TNF-β, and IL-28A. Data were acquired using a Luminex 200 system (Luminex Corp.) and analyzed with xPONENT software (version 4.3.309.1). A minimum of 50 beads per analyte per well was required for data inclusion. Cytokine concentrations were calculated from standard curves generated using a five-parameter logistic (5-PL) regression model. Concentrations were automatically interpolated by the software and expressed in pg/mL. Samples with values below the limit of detection (LOD) were considered negative and assigned a value of 0.00 pg/mL. No samples exceeded the highest standard concentration. Each data point represents an individual mouse. Experimental groups included 3–5 mice per time point.

### Pathological evaluation

The animals were euthanized for necropsy. At the designated study endpoints, mice were euthanized by carbon dioxide (CO_2_) inhalation in accordance with Institutional Animal Care and Use (IACUC) guidelines. No anesthetic agents were administered prior to euthanasia. The lung lobes were collected. Tissues were fixed in 4% paraformaldehyde (Thermo Fisher Scientific, J19943-K2), paraffin-embedded, sectioned at 5-micron thickness and stained with hematoxylin and eosin (H&E) using standard methods. Lung tissues were assessed for pathology by a board-certified veterinary pathologist. The H&E-stained slides were scanned in Zeiss Axio Scan Z1 and the images were analyzed using HALO 4.0 version software (Indica Labs, Inc., Albuquerque, NM, USA). HALO scores served as an indication for the true percentage of lung affected (primary and secondary lesions from TB). The lesions in each lung lobe were scored for pleural thickening, intralobular septae inflammation, perivasculitis, pneumocyte hyperplasia and lymphadenitis. The score was then utilized to annotate the different disease types: active non-necrotizing, active suppurative, active caseous, latent sclerotic, and latent fibrocalcific.

### Immunohistochemistry

Lung tissue was collected during necropsy and fixed in 4% paraformaldehyde (PFA; cat. no. J19943-K2, Thermo Fisher Scientific). Following paraffin embedding, 5 μm-thick sections were cut and mounted on charged slides. Immunohistochemical staining was performed according to the manufacturer’s protocol (Origene). Briefly, slides were baked at 60 °C for 1 hour, followed by sequential deparaffinization in xylene, 100% ethanol, 70% ethanol, and distilled water. Antigen retrieval was conducted using a laboratory-grade microwave in Tris-EDTA buffer (pH 9.0) for 10 minutes at maximum power. After retrieval, slides were washed with Tris-buffered saline with Tween 20 (TBST) and incubated overnight with primary antibodies: anti-hCD4 (1:200 dilution, clone-EPR6855, cat. no. Ab133616) and anti-hCD68 (1:200 dilution, clone- PSH05–47, cat. no. MA5–56511) and anti-hCD163 (1:200 dilution, clone- BLR087G, cat. no. MA5–44365). Sections were then incubated with an alkaline phosphatase (AP)-conjugated secondary antibody (D19–18, Polink-2, Origene), and the signal was visualized using GBI-Permanent Fast Red (supplied with the Polink-2 kit from Origene). All slides were counterstained with hematoxylin (cat. no. CATHE-GL, Biocare Medical) and imaged using a Zeiss Axio Imager Z1 microscope.

### In-situ hybridization

Detection of the *Mtb* organism was performed by RNAScope ISH. Bacilli were detected with 23S rRNA targeted probe sets and the replicative state of the bacilli was assessed by colocalization of pre-rRNA sequence-specific probes, as previously described^26^. Briefly, HIER was performed on tissues with ER2 solution (Leica Biosystems) at 97°C for 30 minutes prior to quenching endogenous peroxidase activity with 3% hydrogen peroxide. *Mtb* was identified using RNAscope 2.5 LS Probes B-MTB-23SrRNA-1-C1 (Advanced Cell Diagnostics #471658) and B-MTB-pre-rRNA-O1-C2 (Advanced Cell Diagnostics # 507548-C2) diluted 1:50 in the C1 probe^27^. Hybridized ISH probes were detected with tyramide signal amplification of Opal 570 to detect 23S rRNA and Opal 690 to detect pre-rRNA. Images were scanned for visualization using an Olympus VS200 scanning microscope with a 60X objective and Z-stack compression. Probes targeting *Mtb* precursor rRNA (pre-rRNA; Opal 690, red), indicative of actively replicating bacilli, and 23S rRNA (Opal 570, yellow), marking total bacterial burden including nonreplicating organisms, were utilized.

### Statistical analysis

Statistical analyses of differences among groups for body weight loss, histological assessment, and immunophenotyping were performed using GraphPad Prism version 10.0 (GraphPad Software, Inc.). All data are presented as the mean ± standard error of the mean (SEM) from at least three independent experiments. Statistical significance was determined using a two tailed Student’s *t*-test or one-way or two-way analysis of variance (ANOVA), as appropriate. A *p*-value of less than 0.05 was considered statistically significant.

## Supplementary Material

This is a list of supplementary files associated with this preprint. Click to download.

- SupplementalFiguresandLegends.docx
- SupplementaryFigures.docx

## Figures and Tables

**Table 1:** Table 1 is not available with this version.

## Data Availability

The datasets generated and/or analyzed during the current study are available from the corresponding author upon reasonable request.
